# The impact of web-based and face-to-face simulation on patient deterioration and patient safety: protocol for a multi-site multi-method design

**DOI:** 10.1186/s12913-016-1683-0

**Published:** 2016-09-07

**Authors:** Simon J. Cooper, Leigh Kinsman, Catherine Chung, Robyn Cant, Jayne Boyle, Loretta Bull, Amanda Cameron, Cliff Connell, Jeong-Ah Kim, Denise McInnes, Angela McKay, Katrina Nankervis, Erika Penz, Thomas Rotter

**Affiliations:** 1 School of Nursing Midwifery and Healthcare, Federation University Australia, Churchill, Victoria 3842 and Mt. Helen, Victoria 3350, Australia; 2 School of Nursing, The University of Tasmania, PO Box 1322, Launceston, Tasmania 7250 Australia; 3 St John of God Health Care Berwick, Gibb St, Berwick, Victoria 3806 Australia; 4 Department of Nursing and Midwifery Education and Strategy, Monash Health, Clayton Rd, Clayton, Victoria 3168 Australia; 5 Latrobe Regional Hospital, 10 Village Ave, Traralgon, VIC 3844 Australia; 6 Nursing and Midwifery, Monash University, McMahons Rd, Frankston, Victoria 3199 Australia; 7 Central Gippsland Health Service, 155 Guthridge Parade, Sale, VIC 3850 Australia; 8 College of Medicine, University of Saskatchewan, Saskatoon, SK S7N 5E5 Canada; 9 College of Pharmacy and Nutrition, University of Saskatchewan, Saskatoon, SK S7N 5E5 Canada

**Keywords:** Clinical audit, Education, Nursing, E-simulation, Patient safety, Simulation-based learning

## Abstract

**Background:**

There are international concerns in relation to the management of patient deterioration which has led to a body of evidence known as the ‘failure to rescue’ literature. Nursing staff are known to miss cues of deterioration and often fail to call for assistance. Medical Emergency Teams (Rapid Response Teams) do improve the management of acutely deteriorating patients, but first responders need the requisite skills to impact on patient safety.

**Methods/design:**

In this study we aim to address these issues in a mixed methods interventional trial with the objective of measuring and comparing the cost and clinical impact of face-to-face and web-based simulation programs on the management of patient deterioration and related patient outcomes. The education programs, known as ‘FIRST^2^ACT’, have been found to have an impact on education and will be tested in four hospitals in the State of Victoria, Australia. Nursing staff will be trained in primary (the first 8 min) responses to emergencies in two medical wards using a face-to-face approach and in two medical wards using a web-based version FIRST^2^ACTWeb. The impact of these interventions will be determined through quantitative and qualitative approaches, cost analyses and patient notes review (time series analyses) to measure quality of care and patient outcomes.

**Discussion:**

In this 18 month study it is hypothesised that both simulation programs will improve the detection and management of deteriorating patients but that the web-based program will have lower total costs. The study will also add to our overall understanding of the utility of simulation approaches in the preparation of nurses working in hospital wards. (ACTRN12616000468426, retrospectively registered 8.4.2016).

## Background

The quality of patient care and patient safety are organisational, individual, and international responsibilities that require an appreciation of adverse events and medical errors [1–3]. In Australia, the Australian Commission on Safety and Quality in Healthcare has prescribed national standards with the objective of improving the quality of clinical care [4]. Health service providers are required to report compliance with these standards, including recognition and response to patients’ ‘severe clinical deterioration’ which is listed as Standard 9 – ‘Clinical Deterioration in Acute Health Care’ [5]. Thus, a key responsibility of providers of acute hospital care, and elsewhere, is to ensure that systems are in place to enable patients with severe clinical deterioration to receive immediate and appropriate assistance [6]. This includes the empowerment of clinicians to activate a ‘Medical Emergency Team’ (MET) (also known as a ‘Rapid Response Team’) whose members are skilled in the management of medical emergencies [7, 8].

Definitions of what constitutes a deteriorating patient or clinical deterioration are lacking, [9, 10] however there is a considerable body of evidence from the ‘failure to rescue’ literature indicating that the management of deterioration can be improved. This includes disturbed physiological variables in the general ward population [11], poorer patient outcomes for mismanaged patient deterioration [12–15], delays in team activation [16], missed indicators of deterioration in rural hospitals [14], a lack of knowledge as to when to call for assistance [17], and a failure to appreciate clinical urgency [18].

Efforts to improve recognition and response to clinical deterioration in Australia include the introduction of a National Patient Observation and Response Chart for recording of patient vital signs and observations [19]. Such standardised reports enable recording of core physiological observations as specified in the National Consensus Statement 1.6 (i.e. respiratory rate, oxygen saturation, heart rate, blood pressure, temperature and level of consciousness). Similarly, in the United Kingdom, physiological track and trigger systems are recommended to monitor all adult patients in acute hospital settings [20]. These charts utilise colour-coded graphed observation and vital sign documentation to designate the level of factors that should trigger an escalation of care. In Australia, the use of one of five available charts is mandatory except for the state of New South Wales where an alternative ‘Between the Flags’ - Standard Adult General Observation Chart is required in most public facilities [19, 21].

In relation to the management of emergencies it is known that the management of deteriorating patients can be enhanced through educative approaches [4, 5, 22–24] and through clinical and simulated experience [25, 26]. Education approaches emphasize the need for active learning in simulated environments that offer authentic learning experiences without risk to real patients [27, 28]. Clinical staff do benefit from simulation-based education in settings which have high fidelity (believability) [26, 29] with consequential reductions in medical errors [30].

This paper describes the protocol for a study that aims to address patient safety in relation to first responders’ ‘failure to rescue’ deteriorating patients, with a focus on enhancing the nursing assessment and management of clinical deterioration. Hospital registered nurse participants will be trained in the requisite primary responses to patient emergencies using two simulation approaches. Participants will participate in either in-situ face-to-face simulation or a web based simulation version of a valid clinical training package: FIRST^2^ACT (Feedback Incorporating Review and Simulation Techniques to Act on Clinical Trends) [31]. A cost benefit analyses will be performed and the clinical impact of training will be determined through a multi-method evaluation that will include quality of care evaluations, primarily achieved through pre-post intervention patient notes review with time series analyses.

## Methods/design

This multi-method study will utilize both qualitative and quantitative research techniques to evaluate the impact of FIRST^2^ACT on the quality of nursing care and patient outcomes. The design is a mixed methods interventional cohort trial comparing the impact of a web-based (Group 1) and face-to-face (Group 2) education program within and between groups.

### Aim and hypothesis

This research aims to measure the impact of two forms of simulation education on nurses’ ability to detect and manage patient deterioration, to assess patient outcomes and to compare the total costs between the two interventions. The research hypotheses to be tested are:

(i). Both the face-to-face and web-based models of simulation education will improve the detection and management of patient deterioration and patient outcomes.
(ii). The web-based program will have lower total costs in improving the detection and management of patient deterioration and patient outcomes.

**Fig. 1:**
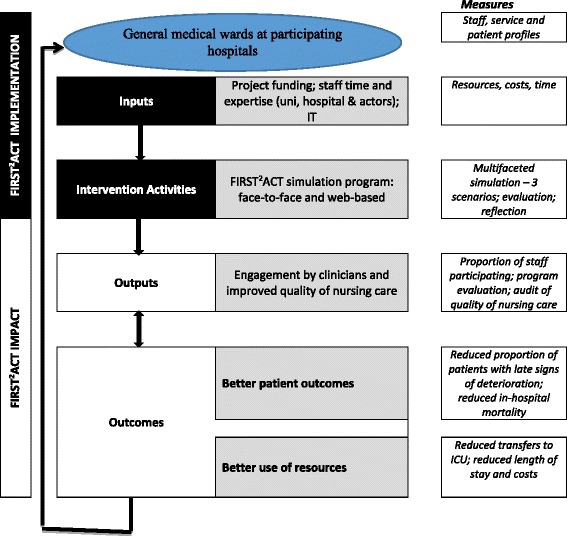
Logic model depicting research structure for the impact of web-based and face-to-face simulation on patient deterioration and patient safety

The primary outcomes to be measured are the proportion of patients showing late clinical signs of deterioration, and the quality of nursing assessment. The secondary outcomes to be measured are the cost of provision of face-to-face versus web-based training, together with stakeholders’ views and attitudes regarding the forms of program delivery, and change to in-hospital mortality and the rate of admission to ICU (see Fig. 1).

### Description of the intervention resource: FIRST^2^ACT

**Fig. 2:**
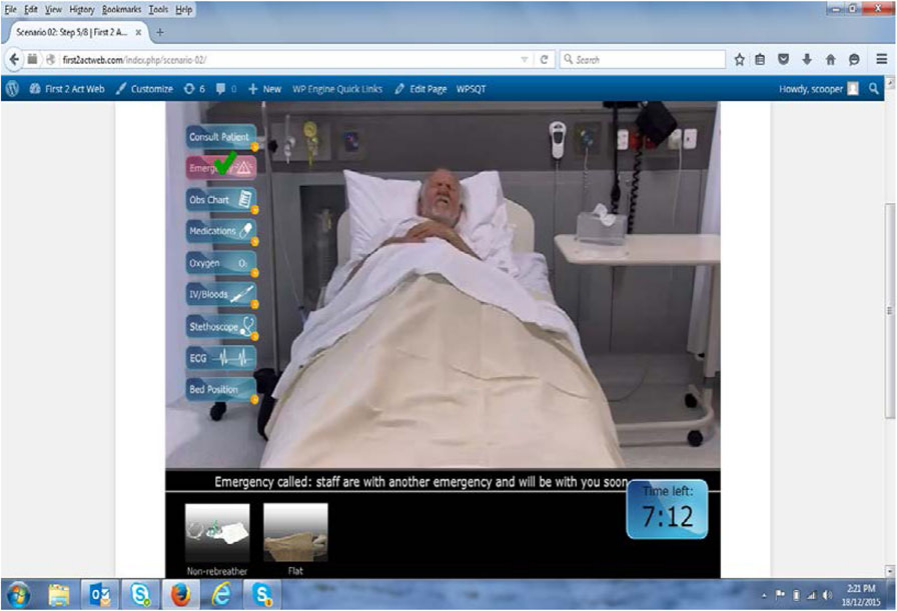
Group 1 - Screen shot of Web based version of First^2^Act interactive patient deterioration education program, depicting patient actor and action buttons

**Fig. 3:**
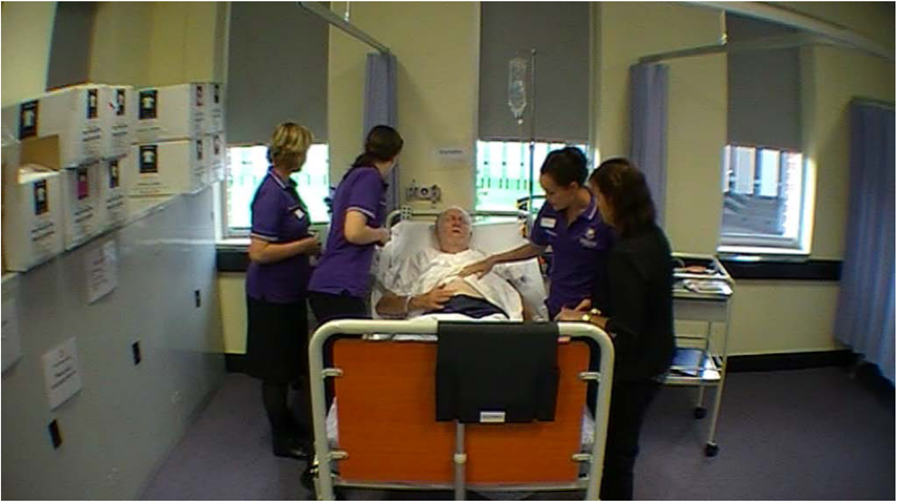
Group 2 - Face-to-face simulation with patient actor during First^2^Act interactive patient deterioration education program

Face-to-face and web based versions of the FIRST^2^ACT program have been developed over the last eight years by some of the authors of this study (SC, RC, LK). The program has been widely reported with notable impacts on learning outcomes and a pilot study that measured the impact on clinical care [29, 32–36]. The web-hosted ‘e-simulation’ version FIRST^2^ACTWeb (see http://first2actweb.com/) (see Fig. 2) incorporates three interactive scenarios (myocardial infarction, shock and respiratory cases) where patient actors deteriorate (over eight minutes) with participants required to ‘click’ on various potential actions – such as measuring blood pressure or inserting an intravenous line - resulting in further pop-up videos of each action. Detailed performance feedback is then provided to a participant on completion [35]. The face-to-face version of the program (Fig. 3) mirrors the web based program (taking between 1.5–2 h to complete) and both include five key components: developing core knowledge; assessment (learning stimulus); simulation; reflective review; and performance feedback [31]. The initial development was funded by the Australian Office of Learning and Teaching from which a full project report is readily accessible [37].

### Study status

This study commenced data collection in February 2016, to be completed by November 2016.

### Sample selection and recruitment

#### Participants

Registered nurses will be recruited from a medical ward in one of four Victorian acute care hospitals. Both Division 1 Registered nurses and Enrolled nurses will be included. Two medical wards will be assigned as Group 1 (web-based intervention), while another two will be assigned as Group 2 (face-to-face intervention). This approach ensures that there is no contamination between homogeneous groups. There are approximately 160 nurses employed at the larger hospitals and 85 at the remaining two sites. In pilot hospital-based studies we have achieved a participation rate of 82 % and anticipate similar participation for this study (*n* = >200). Maximising staff attendance is important in order to have the greatest effect on clinical outcomes.

Nurses in each group will be recruited by a member of the research team who will provide a brief explanation of the study and invite nurses to participate in the study. Participant information sheets and consent forms will be made available and written consent obtained.

#### Clinical impact measures

The impact of the interventions will be assessed on the proportion of patients that progress from early to late signs of deterioration and the quality of nursing care (primary measures). As per our previous studies we will use validated data extraction tools to mine data from patient observation charts. Appropriate frequency of vital signs and evidence-based responses to deterioration will be proxies for the quality of nursing care. Secondary measures include in-hospital mortality and admissions to ICU, and cost of provision of face-to-face versus web based education. We will also investigate stakeholder views and attitudes through semi-structured interviews with three key stakeholder groups at each site.

Patient sample size: Pilot data from an earlier rural study indicated that early signs of deterioration were present in 28 % of patients while late signs were present in 10 %, and that the total sample size required to detect a 5 % reduction in late signs of deterioration with a level of significance of 5 % and a power of 0.8 is 434 patients (217 before and 217 after the intervention) at each site [29].

### Training procedure: participants’ journey

Participating nurses will be released from their working shift for two hours in order to attend the assigned simulation program at their place of work. The procedures for Group 1 web-based intervention and Group 2 face-to-face simulation journey are summarised in Table 1. For Group 2, participants will work in teams of three with a patient actor and research ‘support’ staff; thus over an 8-h shift 12 nurses will complete the training. This release will be enabled by the rostering of additional senior nursing staff as backfill on training days. For Group 1, web-based training will be completed individually on a personal computer or in a ward training room.

**Table 1:** Flow chart of participants’ journey in web-based simulation and face-to-face high fidelity clinical learning environment scenarios

	Group 1	Group 2
	FIRST^2^ACT Web	FIRST^2^ACT In-situ face-to-face
Introductory preamble	Short written introduction with explanation.	Short verbal introduction with explanation.
Demographics and Pre-course MCQ	A demographics form and an 12-item multiple choice questionnaire (MCQ).	As in web-based.
Background material	An animated slide show – with voice over from an educator.	As in web-based.
Scenarios	Scenario (i) – Cardiac scenario Scenario (ii) – Shock scenario Scenario (iii) – Respiratory scenario Total score and feedback provided. Each scenario runs for 8 min with acute deterioration at the mid-point.	As in web–based. Video recorded scenarios are rated by attending researchers.
Feedback	The software gathers all performance data for automated feedback at the end of each scenario.	Face-to-face feedback will be conducted with an educator using video and performance records.
Post-course MCQ	Repeat of the MCQ.	As in web-based.
Course evaluation	A course evaluation and reflective review of educational impact	As in web-based.
Certification	Download a course participation certificate	Issued with a course participation certificate.
Course Manual	Download the course manual for reflection and review	Issued with the course manual for reflection and review
Time commitment	1.5 h	2 h

Described in full below, quantitative data will be captured from the following sources:

1. Patient medical records.
2. A multiple choice knowledge questionnaire (MCQ).
3. Standardized performance measurement forms.
4. Participant evaluations.
5. Costs derived from patient records and inventory of resources.

Qualitative data will be captured in six stakeholder focus groups (three at each site) at the end of the study. Participants will include those who attended training and their line managers in order to elicit the impact of the two programs of education.

### Data collection tools

#### Patient medical records review

Using patients’ demography, diagnoses and vital signs data, outcomes will be measured in relation to deterioration including ICU admissions and mortality rates, etc. Further, based on our sample size calculations and the guidance produced by Harrison et al. [38], McQuillan [39] and NICE [40] we aim to identify a change in patient management by measuring the applicability of physiological observations, actions in relation to deterioration, and early and late signs of deterioration to identify improvements in performance (Table 2). We have developed and tested a chart audit tool based on a rationale for item inclusion, definitions, and clinical expert ratings of relevance and clarity with a high Content Validity Index of 0.83 [41]. We will therefore extract data from patient records from 80 randomly selected cases from each month, three months before the simulation intervention, and from each month for three months after the intervention (total *n* = 480 at each site). A post-hoc sensitivity analysis will be conducted to assess the representative nature of the sample.

**Table 2:** Early and late signs of patient deterioration [Adapted from Harrison et al. [38]

Parameter	Early signs	Late signs
Oxygen saturation	90–95 %	<90 %
Systolic blood pressure (mmHg)	80–100 or 181–240	<80 or >240
Heart rate	40–49 or 121–140	<40 or >140
Respiratory rate	5–9 or 31–40	<5 or >40
Glasgow coma scale	N/A	>2 point drop

### Intervention measures

#### Knowledge

A validated Multiple Choice Questionnaire (pre-post) [42–44] will be completed by participants before and after the program in order to measure patient deterioration knowledge. This measure will enable identification of knowledge in relation to skill performance and changes in knowledge based on program participation.

#### Skill performance measurements

All participants will complete three contrasting simulation exercises that are either recorded as interactive web-based versions or completed face-to-face with a patient actor. Each scenario will be based on common presenting conditions (e.g. acute myocardial infarction, shock and a respiratory case). Standardized patients (actors) will simulate the clinical scenarios. During the simulation exercises, information will be presented in a manner that most clearly reflects the real world requiring participants to be an active searcher [45]. This approach will enhance the ecological validity of the simulation [46] allowing the participant to experience clinical thinking in a more dynamic manner. Levels of relevant information and levels of uncertainty will be taken into account [47] and incorporated into the scenarios.

#### Debriefing and feedback

The web-based version collects performance data for feedback at the end of each scenario, producing a score and written feedback. In the face-to-face version a video recording of the participants’ performance during the simulation exercise enables comprehensive verbal feedback. Written participant evaluations will be sought by survey after the training activities as an evaluation of the educational impact of each program.

### Economic analysis

We will conduct a cost analysis [48, 49] to assess total costs of training. The primary outcome of the cost analysis will be comparison of total costs between web-based versus face-to-face simulation education interventions. Secondary outcomes will include comparison of the costs associated with each intervention (e.g. software development, educator staffing costs, staff release costs, patient actors, etc.). In addition, hospitalization costs will be estimated using the Australian Refined Diagnosis Related Groups (AR-DRGs, Version 7) [50, 51] model, adjusting for ICU admissions and mortality.

### Data analysis

Patient notes review: a pre-post intervention analysis through time series analyses. The appropriateness of observations, incidence of late signs of deterioration, in-hospital mortality and admissions to ICU will be used as the primary measures of patient management. Successful intervention will be identified by a statistically significant increase in applicable actions and a reduction in the proportion of late signs, in-hospital mortality and admissions to ICU.

#### Knowledge and skill performance ratings

Participant demographics, questionnaire, and simulation performance will be described with the use of descriptive and inferential statistics. Measures of dispersion (means, medians, etc.) will be reported. There will be a range of nominal, ordinal and interval data (paired and unpaired) requiring tests such as Chi-square; McNemar; Mann Whitney U; and t-tests. Multivariate analysis (linear and logistic regression) will be used to identify predictors of performance. Simulation exercises will be rated by two researchers for inter-rater reliability. A significance level of *p* < .05 will be applied throughout.

Based upon a Generic Qualitative Design eight stakeholder focus groups will be completed (two at each site) in order to identify the perceived impact of the program(s) [52]. Core themes and outcomes will be identified using Miles and Huberman’s stages of data analysis [53]. Analysts will attribute nominal codes to narrative segments and group narrative segments to identify key themes. Emergent themes will be shared and collaboratively refined to achieve a consensus for reporting. The reporting of findings will by guided by the COREQ guidelines for reporting qualitative research.

The program is designed to run for 18 months with a seven month lead-in, to enable literature reviews, ethical approval, access arrangements and refinement of data collection tools. The training intervention will be compressed into a four month period and subsequent patient notes review, data analyses and report writing will take a further seven months.

## Discussion

This project will add to the body of knowledge in relation to the ‘failure to rescue’ literature with a concern for measuring and improving the management of deteriorating patients. This is a contemporary international issue and one that the project funder – The Victorian Government – is keen to address.

In this mixed methods interventional trial we will investigate the clinical and financial impact of two forms of simulation education. Whilst many studies have identified the educational impact of simulation approaches few have identified how such approaches impact on clinical care and patient outcomes in combination with a cost benefit analyses. Importantly, the reader should note here that it is our intention to compare program *impact* rather than draw direct comparisons between the web-based and face-to-face versions of the program, as they are quite different forms of delivery.

In testing educational interventions Sullivan [54] suggests that there is a need to understand practice and patient safety issues in the real world through ‘pragmatic trials’. These may be cross sectional studies (data from a specified time), longitudinal approaches (exposures over time), cluster randomised trials, quasi (non-randomised) experimental approaches or a range of mixed methods approaches [55]. Individual randomisation of subjects in education trials is problematic as cross-contamination is likely, the dynamics within existing groups will be changed and ethical concerns are raised when individuals are given no choice relating to learning methods [56]. Further, control of such trials is difficult as educational delivery style will differ, participants will attend at different times, and comparison groups are often dissimilar (e.g. comparing lectures with simulation). Randomisation is therefore not the gold standard in education [54, 55].

In large and complex trials of this sort there are a number of issues with a need for effective leadership and management. In this study we have allowed seven months or more to gain ethical approval, access to clinical sites and to develop collaborative networks. The delivery of the simulation interventions will also take time and require careful roll-out in relation to the potentially stressful nature of the training which is designed to elicit the true nature of patient deterioration. Time series analyses and patient notes review is also complex commencing with the challenge of producing a valid and reliable audit tool [41].

However, the project findings will add significantly to our knowledge of organisational systems, adverse events and medical errors. Further, as a result of this multi-method evaluation, we will produce rigorous evidence to inform the development of simulation-based training programs with resultant improvements in patient safety.
